# Jilun Li and his profound impact on theoretical and applied agricultural microbiology

**DOI:** 10.1007/s13238-016-0359-1

**Published:** 2017-01-21

**Authors:** Chengbo Rong, Yu Liu, Ying Wen

**Affiliations:** 1 Institute of Plant and Environment Protection, Beijing Academy of Agriculture and Forestry Sciences, Beijing Engineering Research Center for Edible Mushroom, Beijing, 100097 China; 2 State Key Laboratory of Agrobiotechnology and College of Biological Sciences, China Agricultural University, Beijing, 100193 China

The microbiologist Jilun Li’s excellent work on microbial secondary metabolites and biological nitrogen fixation has made significant contributions to agriculture and has created enormous economic and social benefits in China. He has received many honors throughout his career and was elected as an academician of the Chinese Academy of Sciences in 1995.

**Figure 1:**
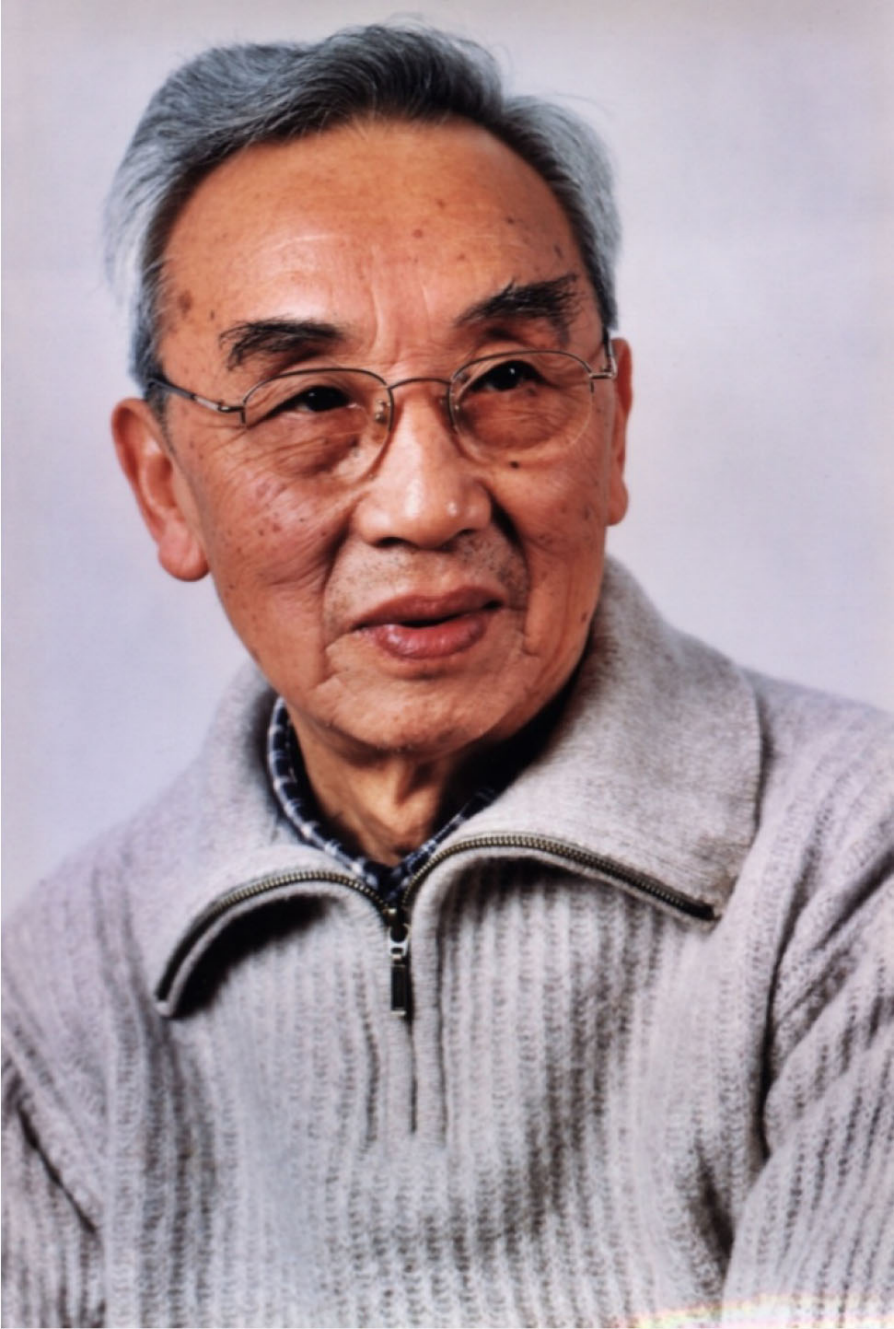
Prof. Jilun Li

Jilun Li was born on March 15, 1925 in Laoting country, Hebei Province, where he was deeply influenced by traditional culture (Fig.1). When he was in fifth grade in primary school, he met the famous scholar Zhaoying Meng, who was a professor at Yenching University. Li had tremendous respect for Prof. Meng and wanted to be a person like him, this belief remained with him all his life. The pursuit of knowledge in his youth was full of hardships, because China was suffering from the war. He risked his life to get through the Japanese blockade, and finally arrived in Chongqing (He 2009). In 1943, he was admitted to the Department of Biology at the National Central University in Chongqing. He graduated and became a teacher at this university in 1948. Then he transferred to the Department of Plant Pathology at the Beijing Agricultural University (now China Agricultural University), and began his scientific research. In 1959, he assisted Prof. Yu to establish the Department of Microbiology, and gradually became a distinguished microbiologist, who developed new applications of microorganisms that greatly benefited agriculture and livestock husbandry.

In 1958, Prof. Li reached a marvelous milestone in his work on gibberellins (GAs), secondary metabolites produced by the pathogenic fungus *Gibberella fujikuroi*, the causative agent of rice bakanae disease, and used as phytohormones in seed germination and fruit development. At that time, GAs were a novel type of phytohormones and were only derived from a few plants abroad. Li and other researchers successfully isolated the GA-producing fungal strain, screened high-yield strains, and optimized fermentation conditions with the guidance of Prof. Dafu Yu (Dafu et al., 1964). The fermentation yield of GAs produced by his *Gibberella* strains reached the world’s advanced level at that period. A GA_3_ crystal weighing 100 g obtained with Li’s strains was displayed at the Leipzig Trade Fair, shocked peer experts from other countries, and won great glory for our country. Thereafter GA_3_ was mass-produced and widely applied in the agricultural field in China. In addition to gibberellin GA_3_, Prof. Li had also great success in the research on other microbial secondary metabolites. In 1989, he successfully fermented and extracted gibberellins GA_4+7_, which had significant impact on increasing the index of fruit-length and overcoming rust disease.

**Figure 2:**
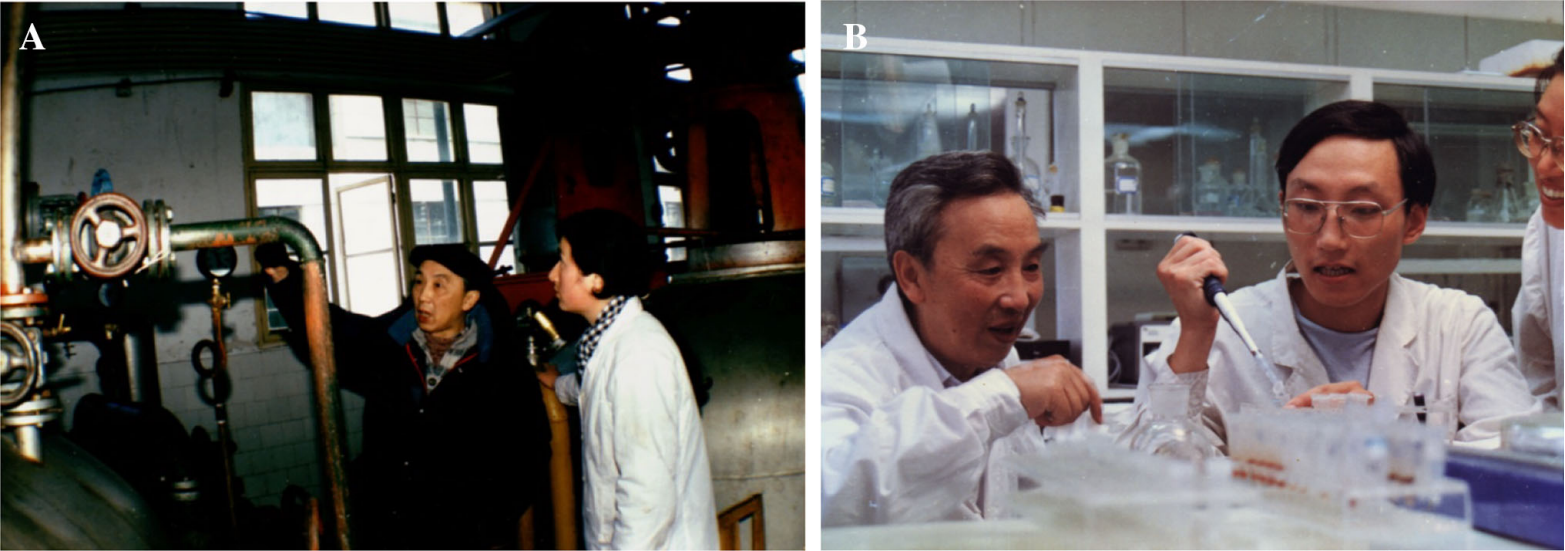
(A) Jilun Li (left) was introducing industrial fermentation technology for a student. (B) Jilun Li (left) worked with students

In addition, Li and his group successfully developed several crucial antibiotics for veterinary and medical use. One example is Chicken coccidiosis, a terrible parasite that causes significant economic losses for the poultry industry. Prof. Li’s group screened high-producing strains for monensin and maduramicin, polyether ionophore antibiotics that have a wide anticoccidial spectrum and good therapeutic effects on coccidiosis even in low dose, established industrial fermentation techniques (Fig. 2A), and made them suitable for mass production by 1985 and 1994, respectively. These accomplishments filled the gap in antibiotic discovery and solved a major problem of the poultry industry in China.

Another example is his outstanding work on avermectins (Li et al., 2010). Avermectins, a series of 16-membered macrocyclic lactones produced by *Streptomyces avermitilis*, are excellent pesticides with high efficiency and low side effects, and are widely used in medical, veterinary and agricultural fields. Avermectin B1a, one of the eight avermectin components, has the highest insecticidal activity. Avermectins were first produced by Merck & Co. which monopolized the international market. In the late 1980s and early 1990s, avermectin B1 (brand name is abamectin) was very expensive, almost 20,000 yuan per kilogram in China. Prof. Li’s group began to study avermectins in 1986. With their persistent efforts, the industrial production of avermectins in the late 1990s in China reached a competitive level and the moderate price forced Merck & Co. to retreat from the Chinese market. Due to this excellent work, Prof. Li and his group won the 2nd prize of the National Science and Technology Progress Award in 2006. In recent years, Li’s group has focused on the elucidation of the complex regulatory network of avermectin biosynthesis and the rational design of new hyperproducer strains through genetic manipulation.

Prof. Li’s research on nitrogen fixation started in the late 1970s. In the summer of 1978, he went to America for the first time to attend the international conference on nitrogen fixation. He was lucky to meet Prof. R.H.Burris from the University of Wisconsin, the leading scientist on the biochemistry research of nitrogen fixation. When he came back from America, Li started the investigation and classification of Rhizobia resources in Xinjiang, which laid the foundation for later research on nitrogen fixation in China. In 1980, he returned to America to study in the laboratory of Prof. Burris at the University of Wisconsin. Prof. Li had been dreaming of studying the catalytic mechanism of nitrogenase for the past twenty years, and he cherished this opportunity very much and worked with great diligence, despite being already fifty-six years old. After two years of hard work, he demonstrated that HD formation is a characteristic of nitrogenase catalysis, and absolutely dependent on N_2_. His findings refuted the hypothesis that HD formation is independent on N_2_ and proved that N_2_H_2_ is the intermediate of the nitrogen fixation process; findings that were widely recognized by the international scientific community. He returned to China in 1982 and continued his research on nitrogen fixation. He proposed a two-site H_2_ evolution model for nitrogenase and already verified it with scientific experiments (Guan et al., 2007). In addition, his group revealed the regulatory mechanism of nitrogen fixation of several nitrogen-fixing microorganisms (Yuan Liu et al., 2003), especially *Azospirillum brasilense*, a root-associated nitrogen fixing bacterium found on members of the Graminae family, including important agronomical crops like wheat, rice and maize. Li’s group engineered an ammonium-resistant strain of *A. brasilense*, which could save 20% of nitrogen fertilizer in maize.

Prof. Li has very extensive research interests, including not only the fields mentioned above, but also the synthesis mechanism and application of other microbial metabolites such as fusarine, zearalenone, poly-β-hydroxyalkanoate (PHA), 1,3-propanediol, astaxanthin and megnetosome.

**Figure 3:**
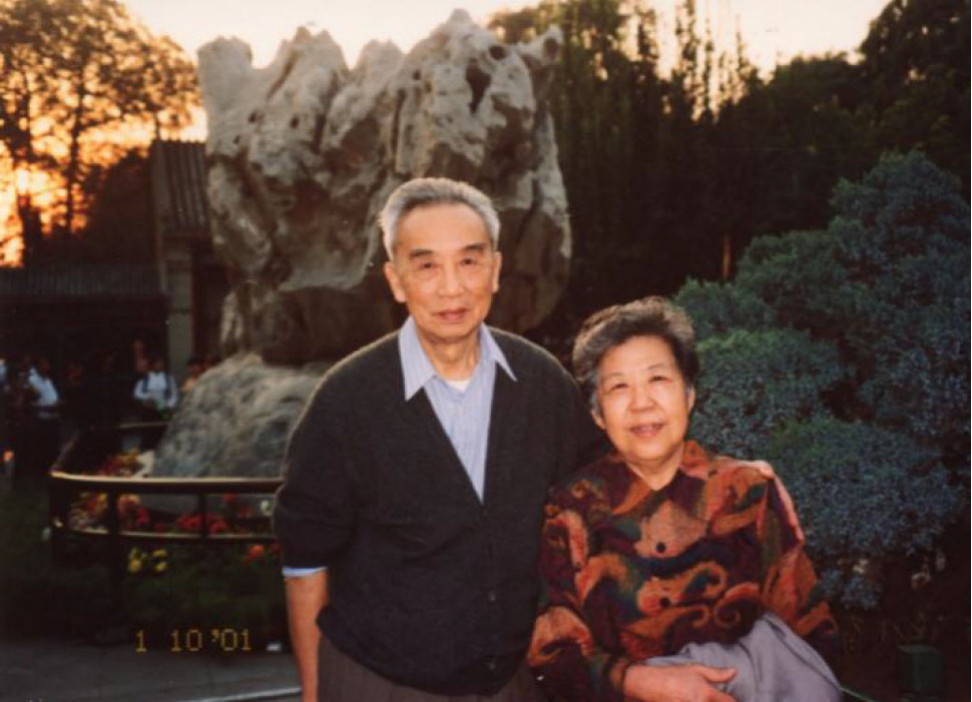
Jilun Li and his wife, Prof. Fanjing Meng

In addition to his great achievements in scientific research, he also devoted his life to teaching and educating students. He has compiled and translated about 4,000,000 words in books, and taught several courses on microbiology. He sets up examples for students with both precept and practice. Prof. Li’s habit of working on experiments together with his students is his most prominent characteristic (Fig. 2B), and he has done this until the age of 80. He is an open-minded, righteous person, always enlightening students to explore the value of life, asking them to work for the need of our country, letting them know the truth that helping others is helping oneself. These teachings will never be forgotten by his students. As his students, we would like to express our deepest gratitude for his contributions and express our devout wishes for his good health and long life (Fig. 3).
